# Laser enhanced photothermal effect of silver nanoparticles synthesized by chemical and green method on Gram-positive and Gram-negative bacteria

**DOI:** 10.1186/s13065-024-01263-7

**Published:** 2024-09-03

**Authors:** Elham M. Mostafa, Y. Badr, Marwa A. Ramadan, Mohamed M. M. Hashem, Khaled Abo-El-Sooud, Heba N. Deif, Amna H. Faid

**Affiliations:** 1 Department of Laser Sciences and Interactions, National Institute of Laser Enhanced Science (NILES), Cairo University, Giza, Egypt; 2 Department of Laser Application in Metrology, Photochemistry, and Agriculture, National Institute for Laser Enhanced Science (NILES), Cairo University, Giza, Egypt; 3 Department of Pharmacology, Faculty of Veterinary Medicine, Cairo University, Giza, Egypt; 4 Microbiology Department, Faculty of Veterinary Medicine, Cairo University, Giza, Egypt

**Keywords:** Antimicrobial blue laser, Bacteria, Green nanotechnology, Multidrug resistance, Nanomedicine, Photothermal agents, Photothermal therapy

## Abstract

**Purpose:**

The antibacterial properties of silver nanoparticles (AgNPs) are extensively identified. In large quantities, they might be harmful. So many fields of nanotechnology have shown a great deal of interest in the development of an environmentally friendly, efficient method for synthesizing metal nanoparticles. Because of its antibacterial and antifungal properties toward a wide range of microbes, chitosan silver nanoparticles (AgNPs@Cs) constitute a newly developing class of bio-nanostructured hybrid materials. Furthermore, the use of photothermal therapy (PTT) has been suggested as a means of elimination of germs. These light-stimulated treatments are minimally invasive and have a few side effects. In the present work, the antibacterial effect of AgNPs at low concentrations; prepared by chemical and green methods as antimicrobial and photothermal agents in photothermal therapy; with laser irradiation were explored as combined treatment against MRSA, *Pseudomonas aeruginosa*, and *Klebsiella pneumoniae*.

**Methods:**

Silver nanoparticles were produced in two ways. First, by sodium borohydrides, second, by chitosan (as a natural eco-friendly reducing, and capping agent). The nanostructure of AgNPs and AgNPs@Cs was confirmed by UV–visible spectrometer, transmission electron microscopy (TEM), Fourier transform infrared spectroscopy (FTIRs), and direct light scattering (DLS). The antibacterial activity of the prepared nanoparticles and the laser irradiation was tested against three bacterial species of zoonotic importance; MRSA, *Pseudomonas aeruginosa*, and *Klebsiella pneumoniae;* and was evaluated by measuring their minimum inhibitory concentrations (MIC).

**Results:**

Silver nanoparticles produced by the two methods had spherical shapes with nearly the same particle size. The analysis of DLS showed that AgNPs were very stable with zeta potential − 28.8 mv, and 47.7 mv by chemical and chitosan synthesis, respectively. Furthermore, AgNPs@Cs showed higher antibacterial activity toward the tested bacterial species than AgNPs by chemical method. Additionally, the bacterial viability using photothermal laser therapy was reduced compared to laser and AgNPs alone. The bactericidal activities were higher when laser diode was coupled with AgNPs@Cs than by chemical reduction.

**Conclusion:**

The laser combined treatment had a higher antimicrobial effect than AgNPs alone or laser irradiation alone.

## Introduction

A major menace to public health is bacterial infection, and despite the effectiveness of conventional antibiotic treatments usually, the widespread and excessive utilization of antibiotics has led to a significant worldwide health crisis in the form of drug-resistant bacteria. Some prevalent microorganisms like *Staphylococcus aureus, Klebsiella pneumoniae, Acinetobacter baumannii* [1], *Pseudomonas aeruginosa*, and *Enterobacter* species can give rise to stubborn hospital-acquired infections, resulting in elevated levels of antibiotic resistance [2, 3]. The inability of antibiotics to effectively combat infections produced by multidrug-resistant pathogens (MDR), particularly those acquired in hospitals, has spurred extensive research to discover alternative antimicrobials that offer greater effectiveness and reduced resistance [4]. The host's immune system is activated to get rid of the invasive microorganisms when an infection arises. But occasionally, severe, and uncontrollable inflammatory reactions can occur, which can result in diseases including multiple organ failure, sepsis, and even death. Therefore, the development of novel methods to attack diverse bacterial diseases is imperative. Drug-resistant bacteria may now be effectively treated using antibacterial nanoparticles as an alternative. They have several exceptional qualities, such as readily changeable surfaces and physicochemical features that may be adjusted, such as shape, particle size, surface charge, and composition. These attributes have great promise for treating many diseases. Many scientists have worked on creating different antibacterial nanomaterials, including metals, silica nanoparticles, metal oxides [5], and nanoenzymes.

Nanoparticles (NPs) are novel, potential medication delivery systems that have shown promise in the treatment of several illnesses [6]. This potency is associated with the capacity to surmount limitations such as limited bioavailability, rapid drug excretion from the body, nonspecific distribution, and poor cellular permeability [7, 8]. In the developing domains of nanomedicine, nanobiotechnology, and nanotoxicology, nanomaterials are more significant. On the other hand, NPs have been used as therapeutic agents against pathogenic microorganisms in the field of toxicity [5, 9–11]. To comprehend the impact of nanoparticles on bacteria, fungi, parasites, and other microorganisms, it is crucial to investigate the characteristics of these particles and how they are used in various biological and medicinal contexts. Additionally, the kind of capping and stabilizing materials that are utilized to prepare NPs is crucial since it influences how effective the NPs are against microbes. They are commonly regarded as a venue for focused drug delivery, clinical diagnostics, and medical imaging. The common inorganic nanoparticles used are olive oil, zinc oxide, silver [12], iron oxide, gold, and titanium. Green nanotechnology as an emerging alternative technology is created on the ecological synthesis of nanoparticles by using the capability of naturally occurring materials to produce various nanoparticles through implementing sustainable processes [7, 10, 13, 14].

When creating safe and useful nanomaterials for medical purposes, the toxicity of silver nanoparticles on live cells must be considered. Ag nanoparticle toxicity to living cells depends on several variables, including the particles' size, shape, surface area, and concentration. Due to their higher surface area and easier penetration of cell membranes, smaller nanoparticles are generally more hazardous than their bigger counterparts. The concentration of Ag nanoparticles can also have an impact on their toxicity to living cells. Higher Ag nanoparticles’ concentrations have the potential to increase toxicity and vice versa. It's unclear exactly how Ag nanoparticles causes harm to living cells. Ag nanoparticles, however, can cause oxidative stress in cells, which can harm lipids, proteins, and DNA. In the end, this may result in cell malfunction or death [15].

For these nanoparticles to effectively limit the bacterial development, they must have a strong synergistic relationship with the biological host. There are now three main pathways. They have been shown to work together or individually to confer antimicrobial activity to AgNPs. First, AgNPs act at the membrane level, as they can cross the outer membrane and accumulate on the inner membrane, where nanoparticle attachment to cells can cause instability and damage, thereby increasing membrane permeability and affecting leakage of cellular contents. And ultimately leads to cell death. Additionally, AgNPs have been shown to interact with sulfur-containing proteins in bacterial cell walls, potentially disrupting the cell wall through structural damage. According to the second mechanism, nanoparticles can enter cells and change their structure and permeability by breaking through the cell membrane. It has been suggested that AgNPs' unique properties will allow them to interact with phosphorus or sulphur groups found in intracellular material, such as DNA and proteins, to modify their structure and functions. Similarly, by reacting with thiol groups in the enzymes that produce reactive oxygen species and free radicals, they can modify the respiratory chain in the inner membrane, causing damage to intracellular machinery and initiating the apoptotic process. The release of silver ions from the nanoparticles is a third process that is suggested to happen concurrently with the first two, which due to their size and charge, they can interact with cellular components and alter metabolic pathways, cell membranes and even genetic material [5, 16]. The progress in nanotechnology has unveiled fresh possibilities in the realm of nanomedicine, enabling the creation of nanoparticles that can be structured into intricate designs. Lately, of blue light has drawn attention to the development of new phototherapeutic antimicrobial agents that exhibit significant antimicrobial activity against a variety of bacterial and fungal pathogens but have a lower probability of resistance compared with antibiotics. Blue light with a wavelength of 400–500 nm has been shown to be very effective against both Gram-negative and Gram-positive bacteria [17]. Photothermal therapy (PTT) is an antibacterial approach derived from thermotherapy, utilizing various laser light sources such as visible, near-infrared (NIR), and ultraviolet (UV) to generate heat [18–20]. PTT relies on the deployment of photothermal agents (PTAs) that generate heat when exposed to electromagnetic radiation, leading to cell membrane disruption, protein denaturation, and irreversible cell damage [19, 20]. When metal nanoparticles (MNPs) or metal oxide Nanoparticles are employed as photothermal agents (PTAs), they induce a phenomenon referred to as “localized surface plasmonic resonance” (LSPR) [9, 21], resulting in elevating of the nanoparticle temperature. The photothermal therapy represents a novel and efficient sterilization technique in which materials transform light energy into heat energy, effectively eliminating planktonic bacteria [22]. Metal nanostructures are the focus of significant interest owing to their distinctive characteristics [9, 21, 23]. Silver nanoparticles (AgNPs) represent a promising biocide, reported to exhibit lower toxicity in comparison with silver ions [13, 24–27]. It is contributed into a variety of antimicrobial applications such as bandages, surface coatings, medical devices, food packaging, functional textiles, and cosmetics [28]. Silver is recognized for its antimicrobial properties against a broad spectrum of microorganisms and has proven a high efficacy in bactericidal applications [25, 29]. Various theories have described the mechanism underlying the antibacterial action of AgNPs, including membrane damage, cellular content leakage, the production of reactive oxygen species, disruption of DNA structure, and enzyme inactivation [30]. Due to the environmental concerns on the risks results from using of chemical-based synthetic methods, applying biological approaches as an appreciated alternatives have become a research hotspot for the biosynthesis of AgNPs [20]. Chitosan (Cs) is a naturally occurring polysaccharide composed of a combination of (1–4)-linked d-glucosamine (deacetylated unit) and *N*-acetyl-d-glucosamine (acetylated unit) building blocks. Extensive research has unveiled that Cs is a natural cationic biopolymer possessing outstanding attributes such as exceptional biocompatibility, biodegradability, non-toxicity, bioactivity, and the presence of multifunctional groups [9, 18, 31, 32]. Owing to its diverse array of exceptional qualities, chitosan finds extensive application in various fields, including food packaging films, bone replacements, artificial skin grafting, biomedical applications, pH-sensitive drug delivery, and numerous other uses [18, 32, 33]. Chitosan hydrogels offer several advantages in various fields. They are commonly used in chemical chemistry and experimental surgery, and for inhibition of matrix proteinases. Additionally, chitosan dressings play a beneficial role in homeostasis and angiogenesis. Chitosan also activates microphages, enhancing their tumor-killing activity and promoting the construction of interleukin-1. Furthermore, chitosan finds applications in biomedical contexts, including wound healing, drug delivery systems, gene delivery, and tissue engineering [32, 34].

The unique physical, chemical, and biological characteristics of silver nanoparticles allow them to engage with bacterial envelopes and prevent bacterial development. Their antibacterial and antiviral properties are attributed to their ability to produce active radicals [15, 27]. Because of the uncontrolled use of antibiotics, many bacterial species developed antimicrobial resistant.

*Pseudomonas aeruginosa* causes diseases in humans and a significant loss in productivity and continues to pose a serious threat to the expansion of this essential business, as well as to the environment and public health. It also creates a range of virulence factors that impact humans and several animal species, including fish [35].

MRSA represents a serious health hazard to both animals and humans. In human, it can cause abscesses and septic wounds [36]. Numerous animal species, including dogs, cats, rabbits, horses, cattle, pigs, and poultry, are affected by MRSA. It can also infect healthy carriers [37].

By rupturing cellular membranes and increasing cellular protein leakage, silver nanoparticles (AgNPs) exhibit remarkable antimicrobial properties against *Klebsiella pneumoniae*. This also reduces the production of extracellular polymeric substances (EPS), which are essential for the formation of biofilms, thereby inhibiting *K. pneumoniae*’s ability to form biofilms. AgNPs cause multi-drug-resistant *K. pneumoniae* to exhibit an antibacterial action mechanism similar to that of triclosan [38].

In the present study, we conducted a comparative investigation using both chemically synthesized and green-synthesized silver nanoparticles as photothermal agents for photothermal therapy towards Gram-positive and Gram-negative bacteria of medical importance.

## Materials and methods

### Preparation of silver nanoparticles (AgNPs)

Silver nanoparticles were prepared by two different methods. The first one is chemically by using sodium borohydride reduction, and the second method is biosynthesis via chitosan as a reducing and a capping agent.

### Synthesis of AgNPs by a modified chemical method

The synthesis of AgNPs was done according to the chemical reduction methods described by [20, 25, 27, 39], with a slight modification to get smaller particles with narrower size distribution. Briefly, a mixture of 0.08 gm of trisodium citrate and 0.2 g PVP, has been dissolved in 10 ml water, 0.127 g silver nitrate was dissolved in 75 ml of distilled water, then added to the above mixture with continuous steering for 15 min. An amount of 0.0378 g of sodium borohydride (the reducing agent) dissolved in 10 ml cold distilled water was added quickly to the mixture, with continuous stirring for approximately 20 min. Then, the alteration of silver nitrate to silver nanoparticles was specified by varying the solution color to dark brown.

### Synthesis of AgNPs@Cs by chitosan reduction

In the present experiment, the synthesis of AgNPs was done according to the method described by [40], with a slight modification to obtain a narrower size distribution. Briefly, 1 g of medium molecular weight chitosan was dissolved in a 1% acetic acid (v/v) solution. Eight ml silver nitrate solution (8.89 mg/ml) was mixed with 20 ml of chitosan solution (6.92 mg/ml) and stirred for 30 min until being homogenous. Next, the above mixture was transferred to a beaker, and left at 95 °C for 12 h. Within, few hours after the reaction, the solution changed from colorless to light yellow and finally, to yellowish brown color, indicating the formation of AgNPs.

### Characterization of AgNPs

#### UV–Vis spectroscopy measurements

That was recorded via Cary 5000 (UV–Vis spectrophotometer, Varian) in a wavelength ranging from 200 to 800 nm.

#### Transmission electron microscope (TEM)

A high-resolution transmission electron microscope (HR-TEM, Tecnai G20, FEI, Netherlands) was used for imaging. A drop of diluted sample solution was placed on a copper grid coated with amorphous carbon. Bright-field imaging mode was done with an Eagle CCD camera using a lanthanum hexaboride (LaB6) electron source gun and an electron acceleration voltage of 200 kV.

#### Dynamic light scattering

The measurements were performed via a Zeta-Sizer Nano-ZS (Malvern Instruments Ltd, UK) equipped with a 633 nm laser. This analysis provides the size distribution and polydispersity index (PDI) of particles within the sample. The hydrodynamic particle diameter can be obtained by the Stokes–Einstein equation.

#### Fourier transform infrared spectroscopy (FTIRs)

FTIRs were performed between 500 and 4000 cm^−1^ via an FTIR spectrometer (4100 Jasco, Japan), by means of a lyophilizer, AgNPs, and AgNPs@Cs were freeze-dried.

### Bacterial cultures

The bacterial cultures used in this study are three bacterial species of zoonotic importance [Gram positive (MRSA), and Gram-negative bacteria (*Pseudomonas aeruginosa* and *Klebsiella pneumoniae*)]. Two of the tested bacterial species are American Type Culture Collection; MRSA (ATCC43300), and *Pseudomonas aeruginosa* (ATCC9027); and *Klebsiella pneumoniae* strain, which was isolated from chicken meat, identified, and deposited in the culture collection of Ain Shams University as (CCASU-2022-44).

#### *Minimum inhibitory concentration (MIC) and minimum bacterial concentration (MBC) *[41]

For all biological assays, the plate count method was used to control the minimum inhibitory concentration (MIC) of the AgNPs and AgNPs@Cs on the tested bacteria growth. Each tested strain was grown overnight aerobically on Brain Heart Agar (MHA, Difco, France) at 37 °C. A single colony of each bacterial species was picked up and inoculated in Muller-Hinton broth (MHB) medium. Pure bacteria cultures were grown overnight and then adjusted to a concentration of 1 × 10^8^ CFU/ml using the 0.5 McFarland standard. Then, the bacterial strains were diluted to approximately 10^6^ colony-forming units per ml (CFU. ml^−1^). In a 96-well microliter plate, AgNPs and AgNPs@Cs solutions (100 μl of 97 and 78.4 µg/ml, respectively) were double fold serially diluted with sterile distilled water before being pipetted into wells No. 1 through No. 12 of column A. The following step involved adding 100 μl of each tested bacterial culture to well No. A1 such that the total volume was 200 μl and the concentrations for AgNPs and AgNPs@Cs solution were 48.5 and 39.2/ml, respectively. Well No. A11 was used as a negative control containing only Ag NPs, while the well No. A12 was used as a positive control containing only the bacterial culture. These procedures were carried out for every bacterial species. After covering the plates, they were incubated in a shaker incubator for 20 min at 37 °C then, 30 μl from each well was spread on Mueller–Hinton agar plates (10 μl/plate) and incubated at 37 °C for 24 h to determine the bactericidal effects. The lowest concentration of AgNPs and AgNPs@Cs in the sequences preventing the progress of the bacteria in vitro was measured as the MIC.

#### In vitro laser treatment

For laser irradiation, a DPSS laser with a wavelength of 405 nm and 150 mW was used. Overnight cultured bacteria were collected and resuspended in fresh MHB to a final concentration of 10^8^ CFU/ml. A total of 100 μl of each bacterial culture was transferred to a 96-well plate, and the irradiation was done in a time series (0, 2, 4, 6, 8, 10, 12, 14, 16, 18, and 20 min). After irradiation, 30 μl from each well was spread on Mueller–Hinton agar plates (10 μl/ plate) and incubated at 37 °C for 24 h to test the inhibitory effect of the laser treatment.

#### In vitro photothermal treatment

To test the enhancing efficiency of laser on AgNPs and AgNPs@Cs, the cultured bacteria were collected separately, and resuspended in fresh MHB broth to a final concentration of 10^8^ CFU/ml. A total of 100 μl of the bacterial culture was treated; separately; with the lowest concentration of silver nanoparticles that was previously recorded to decrease the bacterial growth, were transferred into a 96-well plate and each test bacteria was incubated with AgNPs and AgNPs@Cs, independently for 20 min irradiated with a time series (0, 2, 4, 6, and 8 min). After irradiation, 10 μl from each well was spread on Mueller–Hinton agar plates (MHA) and incubated at 37 °C for 24 h to evaluate the combined effect of both treatments.

### Statistical analysis

The statistical analysis was carried out using R software version R-4.3.2. The analysis was carried out to evaluate the effect of the photo-thermal therapy, nanoparticles, and the combined effect of both photo-thermal therapy and the nanoparticles.

## Results and discussion

### UV–VIS absorption and TEM of AgNPs and AgNPs@Cs

**Fig. 1:**
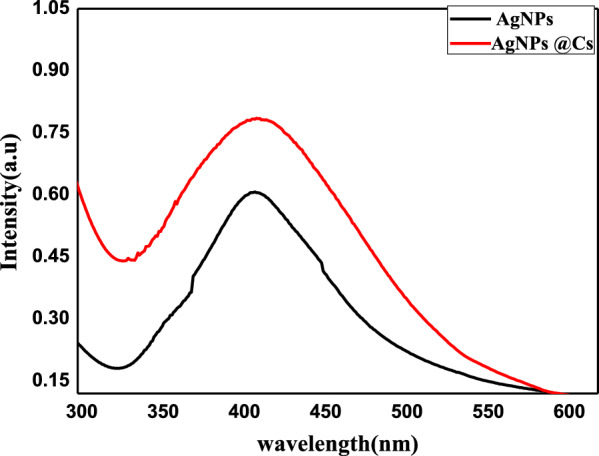
UV–visible absorption spectra of AgNPs and AgNPs@Cs

The UV–VIS absorption spectrum exhibits the representative Surface Plasmon Resonance (SPR) band of AgNPs, centered at approximately 410 nm as shown in Fig. 1 signifying the formation of AgNPs, and indicating that the prepared AgNPs by the two methods had spherical shape and slightly of the same size. In addition, the SPR band of AgNPs by chitosan was more broadening with a slight increase in its intensity compared with AgNPs by chemical method. That may be due to the polymeric stabilization of chitosan on AgNPs compared to steric stabilization of AgNPs by the chemical method. Additionally, the concentration of the obtained AgNPs by chitosan is slightly more than that by chemical method. Surface Plasmon Resonance (SPR) bands were detected at 410 nm, which approves the formation of AgNPs in chitosan. Chitosan is an extremely powerful polymeric chelating agent for silver ions because its amino groups have a significant affinity to form a complex with metal ions. In the current system, the complex of silver ions with amine groups in chitosan, chitosan’s reduction of the metal precursor, and incorporation of the resultant silver nanoparticles into a biopolymeric matrix formed by chitosan's oxidation all led to the spontaneous generation of silver-impregnated chitosan [42].

**Fig. 2:**
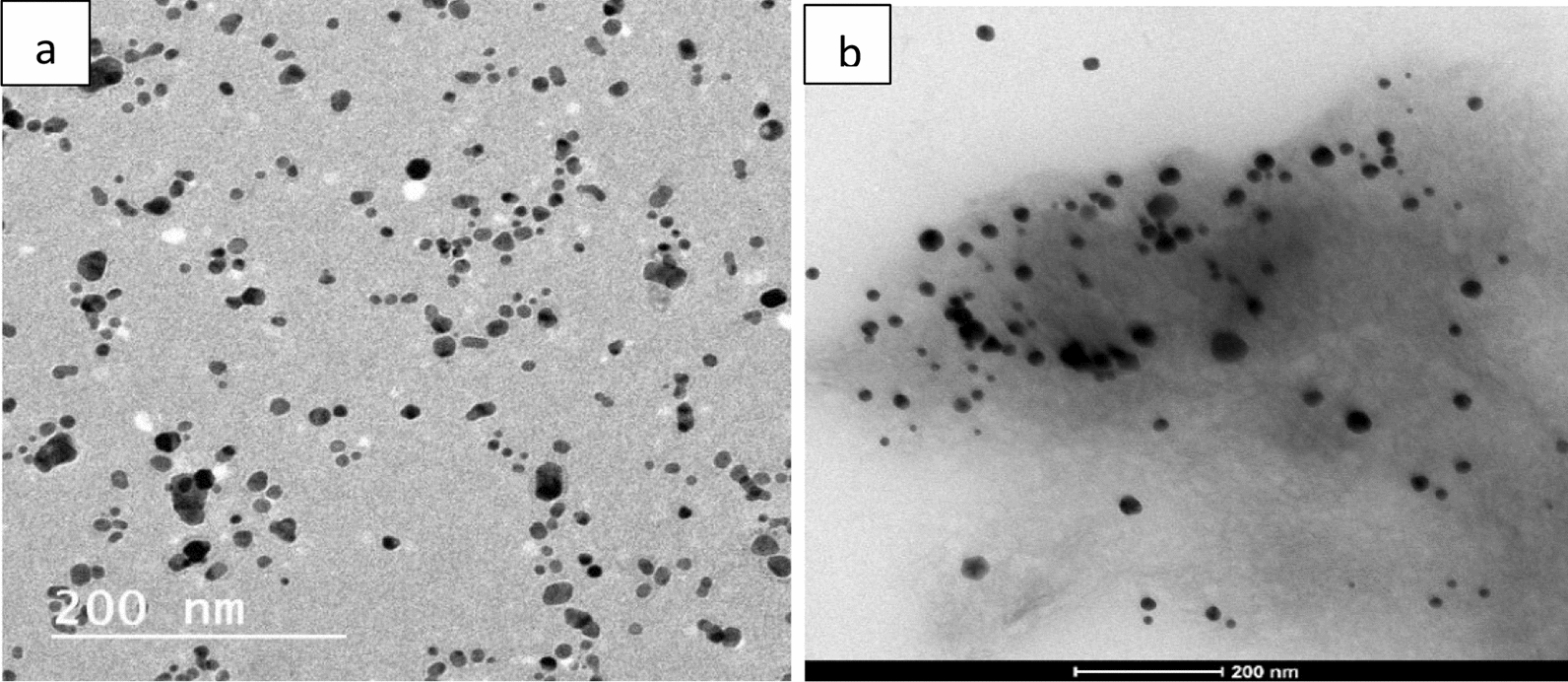
Transmission electron microscopy images of the size distributions of **a** AgNPs and **b** AgNPs@Cs

The TEM images are demonstrated in Fig. 2a, b. These images show that the prepared AgNPs and AgNPs@Cs were spherical with small size distribution of approximately 10 ± 2 nm and 13 ± 2 nm, respectively with little to no aggregation between the synthesized AgNPs and AgNPs@Cs, which implies the repulsion of the prepared nanoparticles to avoid aggregation by steric and polymeric stabilization, respectively.

### Zeta-potential and particle size distribution using dynamic light scattering (DLS)

**Fig. 3:**
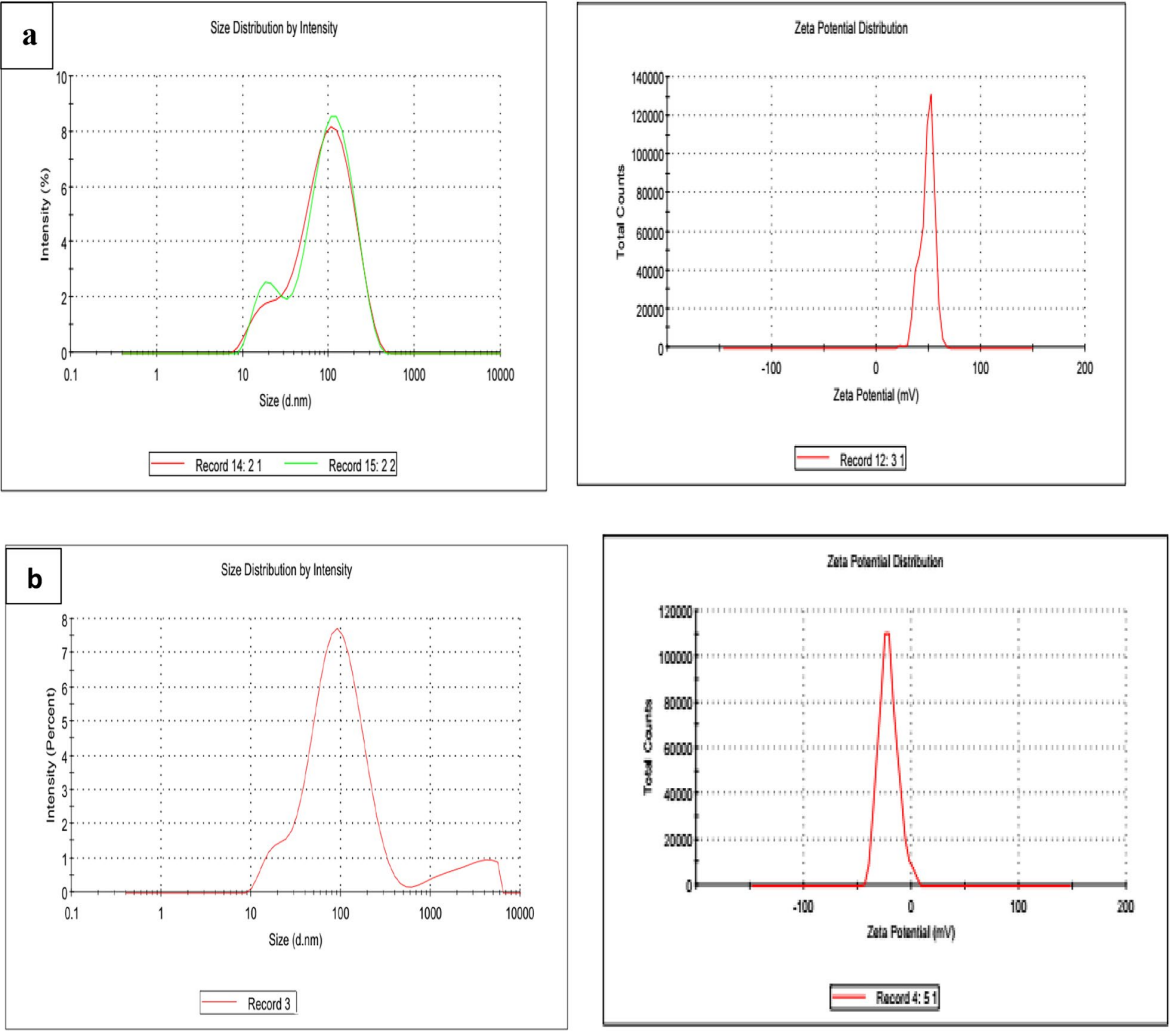
The size distribution and zeta potential of **a** AgNPs@Cs and **b** AgNPs

Direct measurements of light scattering and zeta potential were conducted to evaluate the hydrodynamic size and the surface charge of the colloidal AgNPs and AgNPs@Cs. Typically, nanoparticles with zeta potentials exceeding + 20 mV or falling below − 20 mV demonstrate enough electrostatic repulsion among similarly charged particles, ensuring their stability [19]. The results for the AgNPs’ particle size and zeta potential were as follows: the AgNPs@Cs exhibited a particle size of 123.5 nm and a zeta potential of 47.7 mV. In contrast, AgNPs exhibited a particle size of 105.9 nm and a zeta potential of − 28 mV, as shown in Fig. 3 and Table 1. It’s important to note that the hydrodynamic size is larger than what TEM characterization revealed, likely due to the presence of hydrated molecules surrounding the water-soluble AgNPs’ core. The zeta potential serves as a pointer of stability for nanoparticles. The negative zeta potential value observed for the AgNPs synthesized through citrate reduction (− 28 mV) can be attributed to the existence of three deprotonated anionic carboxyl groups from citrate ions, promoting repulsive interactions among nanoparticles and effectively preventing AgNPs agglomeration. The table also indicates that the Polydispersity Index (PdI) values for AgNPs produced via citrate and chitosan reductions were 0.3 and 0.4, respectively. These values indicated the homogeneity of the resulting particles, with citrate synthesized AgNPs tending to exhibit slightly greater homogeneity.

**Table 1:** The particle size (nm), and zeta potential of AgNPs by citrate, and chitosan reduction

AgNPs	Average particle size (nm)	PDI	Zeta potential (mV)
AgNPs	105.9	0.308	− 28.8 mv
AgNPs@Cs	123.5	0.460	47.7 mv

### Chemical bond composition via FTIR

**Fig. 4:**
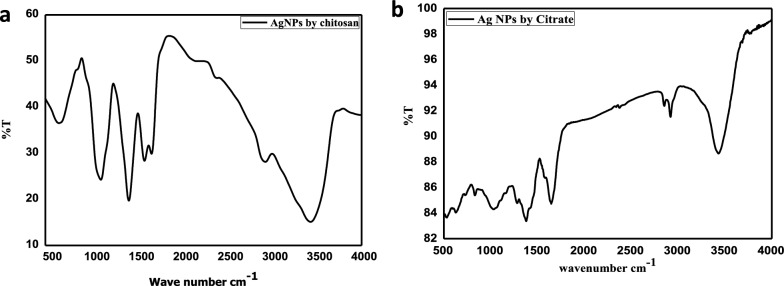
FTIRs of **a** AgNPs@Cs, **b** AgNPs

FTIR analysis detecting the various functional groups responsible for reducing Ag+ ions and capping/stabilizing agents. As depicted in Fig. 4a, the spectra of AgNPs@Cs solutions exhibited distinctive bands associated with the reduction and capping of silver ions by chitosan. The AgNPs@CS spectrum revealed a band at 3442 cm^−1^, corresponding to the N–H stretching of the amino group, while the bands observed around the 2922 cm^−1^ region indicated the C–H and C–H stretching vibrations of aromatic compounds. Moreover, the bands at 1635 cm^−1^ correlated with (NH) C=O stretching vibrations. Additionally, the band at 1530 cm^−1^ were corresponded to the binding vibrations of amide I and amide II, respectively. Furthermore, the bands at 1460 and 1057 cm^−1^ were assigned to N–H and C–N stretch vibrations of amines, while the band at 652 cm^−1^ corresponded to the C–H bending vibrations of stabilized silver nanoparticles [43, 44]. The presence of N–H, and C–N absorptions may be attributed to result from the interaction between amino groups and the metallic surface of AgNPs, where the amino groups performed as capping agents to stabilize AgNPs@Cs [43].

FTIR spectra of AgNPs were revealed in Fig. 4b. The peaks at 1078 cm^−1^ for –C=O stretch for carboxylic acid and 1850 cm^−1^ –C–OH stretch assigned for carboxylic acid from tri-sodium citrate as capping agent [45]. The absorption peak at 1378 cm^−1^ were corresponding to the NO_2_ compound [46], and the bands at 3429 and 2919 cm^−1^ are assigned to O–H and C–H stretches, respectively. The band at 832 cm^−1^ is attributed to C=C bending alkene, the C=O groups of pure PVP showed a prominent peak at 1646 cm^−1^. These characteristic bands can be investigated to explore the interaction between PVP and Ag metal ions [47].

### The effect of AgNPs and AgNPs@Cs on the tested bacterial strains

**Fig. 5:**
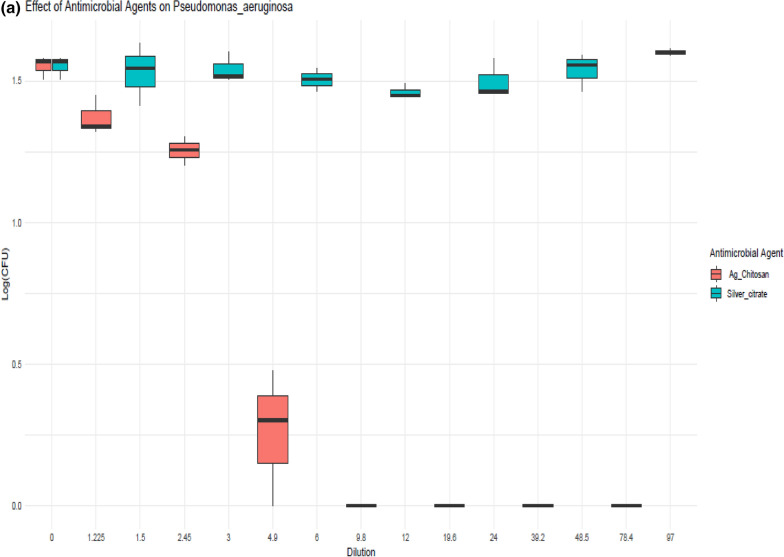

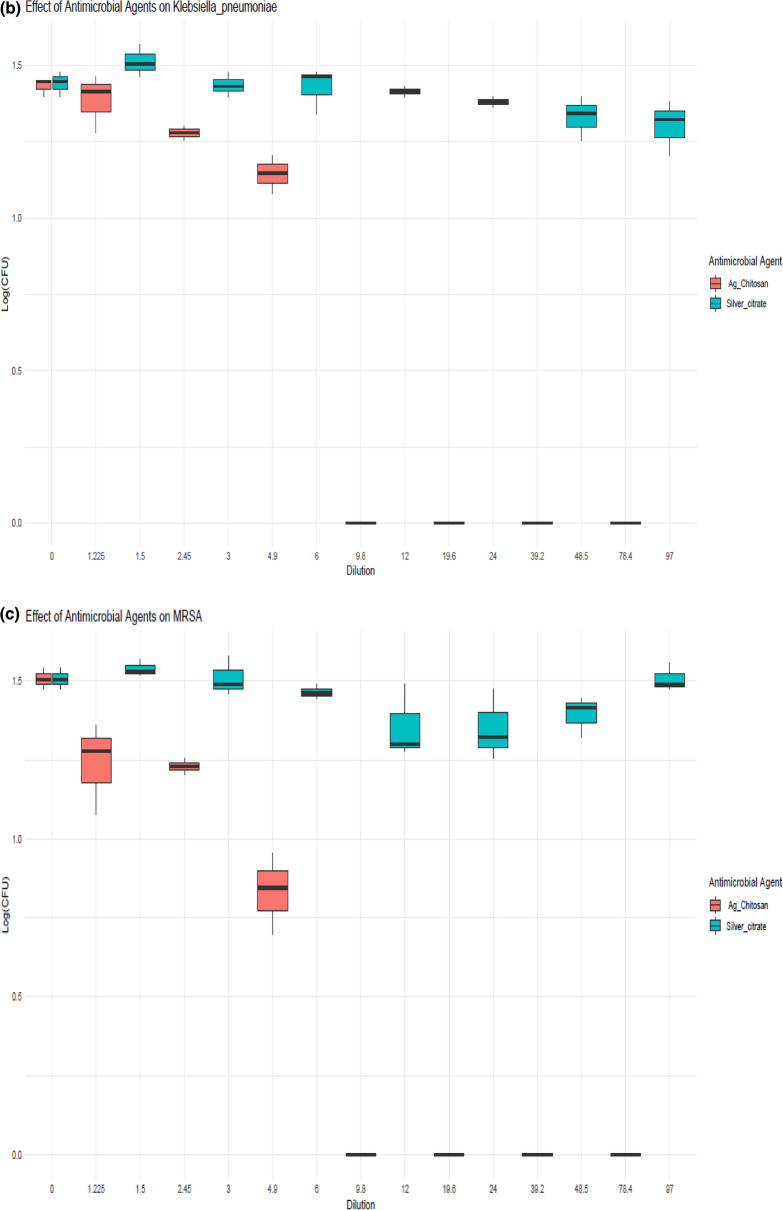
Viability growth inhibition at different concentrations of Ag NPs by citrate and AgNPs@Cs on** a** *Pseudomonas aeruginosa*** b*** Klebsiella pneumoniae* and** c** MRSA

The inhibition effect for different concertations of AgNPs and AgNPs@Cs (1.5, 3, 6, 12, 24 and 48.5 µg/ml) and (0.6, 1.2, 2.4, 4.9, 9.8, 19.6, and 39 µg/ml) on MRSA, *Pseudomonas aeruginosa*, and *Klebsiella pneumoniae* are shown in Fig. 5. The results show a reduction in viability reached (0, 0, 9.2, 28, 29, 22.6 and 0%), (1, 2, 10.7, 18.5, 10.2, 2.5 and 0%), and (0, 1.2, 2.4, 6, 13.3, 21.7 and 26.5%) on MRSA, *Pseudomonas aeruginosa*, and *Klebsiella pneumoniae*, respectively. Additionally, there was a variation in the microbial reduction with AgNPs by chemical method as the highest concentration didn’t induce the highest inhibition, as the maximum inhibition concentration was (12,6 and 48.5 µg/ml) on MRSA, *Pseudomonas aeruginosa*, and *Klebsiella pneumoniae,* while, AgNPs prepared by chitosan results show a reduction in viability which reached (35, 47, 78, 100, 100, 100 and 100%), (33.6, 49.5, 94, 100, 100, 100 and 100%), and (11, 31.3, 49.4, 100, 100, 100 and 100%) on MRSA, *Pseudomonas aeruginosa*, and *Klebsiella pneumoniae,* respectively.

Numerous investigations have demonstrated the antibacterial action of AgNPs. Others have demonstrated the mechanism of their contact with bacterial cells. AgNPs; according to one theory; are just sources of silver ions, which they deposit onto the cell wall where they attach to the cytoplasmic membrane [48]. Silver ions are thought to react with the thiol groups of protein molecules, disrupting enzyme function and sometimes resulting in cell death. Figure 5 illustrates how AgNPs themselves have a greater antibacterial action. Because AgNPs are bigger than silver ions. For that, AgNPs are prepared to get more surface area, consequently, interact with more molecules and thus exhibit greater antimicrobial activity [49]. As shown in Fig. 5, in all tested concretions of AgNPs@Cs, there was a significant reduction in viability on the three tested microbes which may be due to that through a variety of methods. Chitosan can prevent the growth of bacteria. The most basic one is an electrostatic contact between the positively charged NH^3+^ sites of chitosan and the negatively charged membranes of microorganisms. Intracellular material is released because of the contact changing the microbial cell permeability. It has also been shown that chitosan is an efficient way to change the calcium content of cell walls. In this instance, chitosan alters the osmotic equilibrium of the membrane wall and interacts with the stability of peptidoglycan to provide its antibacterial action. Chitosan can also affect the electron transport chain and oxygen reduction mechanisms, which can compromise the membrane’s energy stability. Another proposed mechanism is related to the ability of chitosan to cheat metal ions, as it disrupts the integrity of microbial membranes. Positively charged chitosan can also block RNA and protein synthesis, thereby inhibiting bacterial growth. The antibacterial characteristics of AgNPs decreased with chitosan may provide an explanation for this phenomenon. First, it is thought that electrostatic contact accounts for most of the interaction between bacterial cells and chitosan. The protonated amino group of chitosan's positive charge interacts with the negatively charged molecules on the bacterial cell surface, causing the cell surface to be permeable, which in turn allows internal chemicals to seep out and ultimately results in the death of the bacterial cell. Furthermore, it has been reported that the antibacterial mechanism of AgNPs@Cs results in alterations to the membrane potential and permeability. These modifications upset the homeostasis of the cell and ultimately cause bacterial cell death. This could account for the greater inhibition effect of AgNPs@Cs when compared to AgNPs through chemical reduction [50].

### Effect of laser irradiation on the tested bacteria

**Fig. 6:**
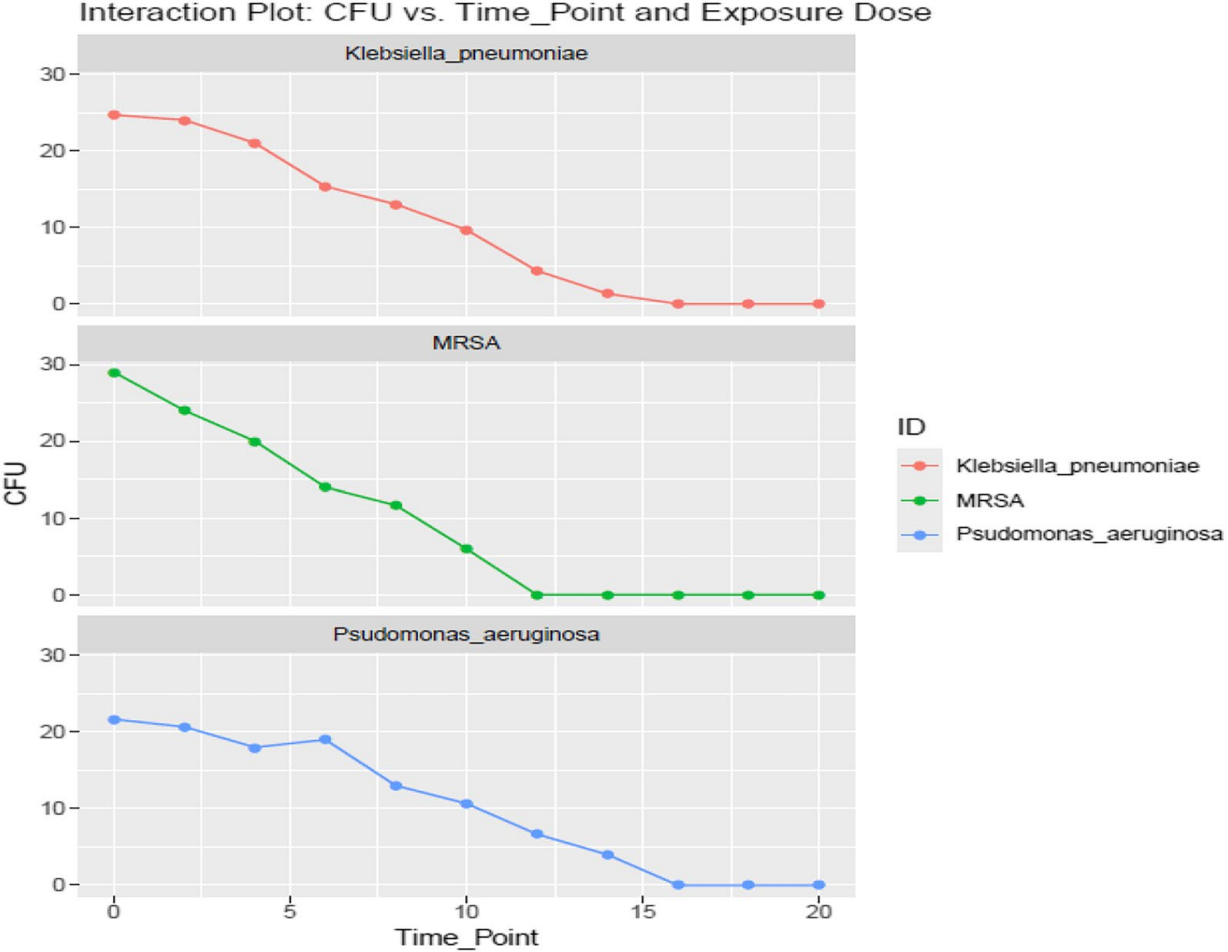
Laser irradiation effect on the tested bacterial species

Calculating the CFU of the tested bacteria before and after laser treatment allows one to assess the inhibitory effect of laser light (405 nm) on MRSA, *Pseudomonas aeruginosa*, and *Klebsiella pneumoniae* for 2, 4, 6, 8, 10, 12, 14, 16, 18 and 20 min, as represented in Fig. 6. MBC were reached at 12, 16 and 16 min which are equivalent to the exposure doses 262.8, 350 and 350 J/cm^2^, while MIC was 10, 14, and 14 min which is equivalent to the exposure doses 219, 306.6 and 306.6 J/cm^2^ for MRSA, *Pseudomonas aeruginosa*, and *Klebsiella pneumoniae*, respectively.

### Assessment of photothermal effect on the tested bacteria

**Fig. 7:**
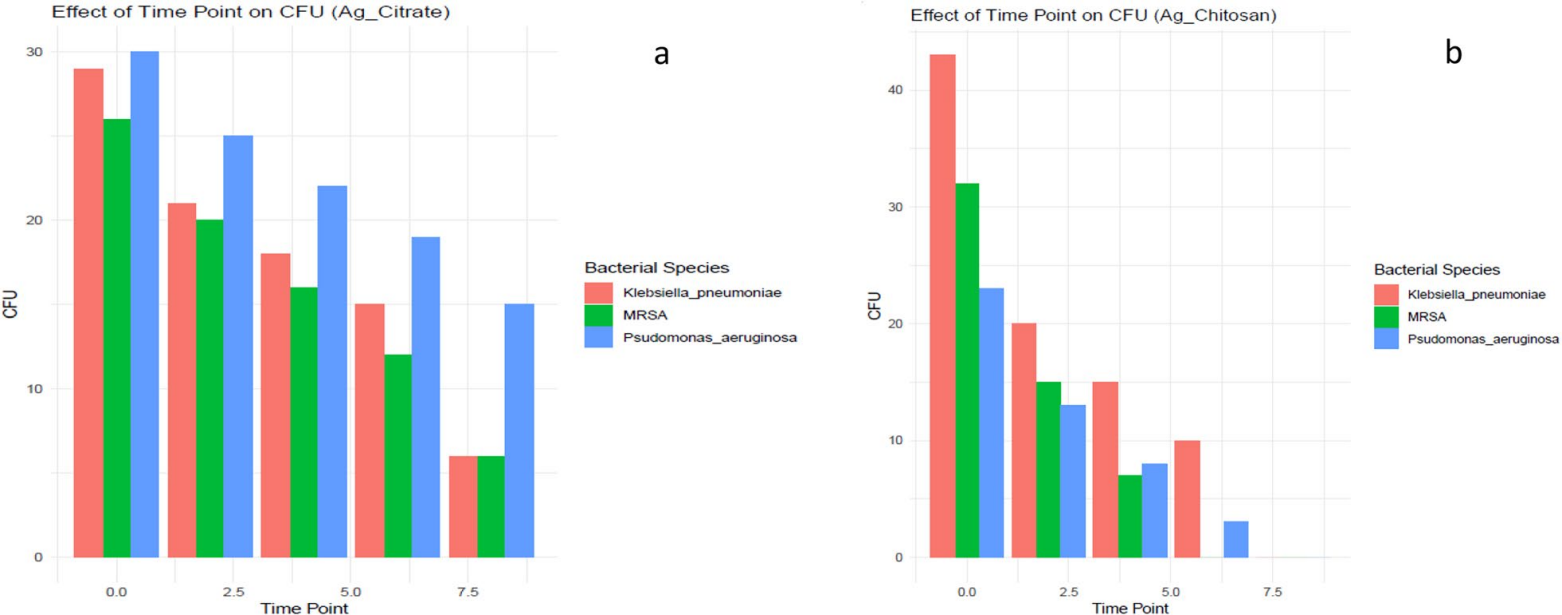
Photothermal effect by **a** AgNPs & **b** AgNPs@Cs

The growth pattern of MRSA, *Pseudomonas aeruginosa*, and *Klebsiella pneumoniae* in the presence of AgNPs and AgNPs@Cs with 405 nm (150 mW) laser irradiation are exposed in Fig. 7a, b, respectively. It was carried out to test the photothermal effect against the three tested bacterial species. In this step, we used the safe lowest concentration (1.5 and 1.225 µg/ml) for AgNPs and AgNPs@Cs, respectively and irradiated for 0, 2, 4, 6, 8 min with 405 nm laser light. As shown in Fig. 7, there was a significant reduction in viability with increasing irradiation time, in case of AgNPs by chemical method irradiated by laser for 8 min resulted in a reduction reached (78.5, 69 and 68%) on MRSA, *Pseudomonas aeruginosa*, and *Klebsiella pneumoniae,* respectively. While, in AgNPs@Cs irradiated with the same laser light reached MBC at 6, 8 and 8 min and MIC at 4, 6 and 6 min which is equivalent to the growth inhibition of 72, 88 and 79% on MRSA, *Pseudomonas aeruginosa*, and *Klebsiella pneumoniae*, respectively.

Whatever the particle size or laser energy, combining the use of laser and AgNPs further inhibited the development of bacteria, suggesting that the combined treatment of laser and AgNPs is superior to either therapy alone. These bacterial inactivation methods may help to explain our findings that 405 nm laser irradiation is antimicrobial and that laser therapy with AgNPs treatment has even more potent antimicrobial activity than laser treatment alone [18, 51]. ROS are known to cause metabolic pathway disruption, which leads to cell malfunction. AgNPs also can adhere to cell membranes and alter the chemical and physical makeup of cell walls, which can hinder and interfere with essential cellular processes like respiration, ion transport, cell division, and membrane permeability [52].

After testing the effect of the laser light on the bacterial growth, and viability, it was clear that there was a statistically significant effect of the photo-thermal therapy by time on bacterial CFU levels across the three tested bacterial species (the p-value for the “Timepoint” factor is 0.00304, which represents the significance of the time effect. These results agreed with that mentioned by [53] who suggested that laser treatment can increase the antimicrobial efficacy of MRSA. The effective laser exposure time for MRSA was 12 min but the effective one reported by [54] was 5–6 min. This is also compatible with [55] who confirmed that the laser therapy was very effective in killing of *Pseudomonas aeruginosa.*

In the present study, photothermal therapy is effective in killing MRSA, *Klebsiella pneumoniae*, and *Pseudomonas aeruginosa*. The effective exposure times to the laser therapy for MRSA, *Pseudomonas aeruginosa, and Klebsiella pneumoniae*, were 12, 16, and 16 min, respectively. There were distinct differences among these three bacterial species, as when AgNPs were tested, it couldn’t inhibit the bacterial growth of any of the tested bacterial species. Its starting concentration was 48.575 µg/ml, which then was serially diluted to 1.5 µg/ml. So, AgNPs alone were not effective, but [56] stated that AgNPs effectively inhibited the growth of MRSA, and K*lebsiella pneumoniae*, which may be attributed to the difference in the used concentrations. Conversely, the ANOVA outcomes presented the statistical significance of the difference in mean CFU counts between different groups (for each bacterial species, the AgNPs@Cs, dilution, and AgNPs@Cs, dilution). The p-value for each term indicated whether there was a significant change in mean CFU counts between different groups. For MRSA, the p-value was significant for AgNPs@Cs (p = 0.000435); meaning that AgNPs@Cs had a significant effect on the bacterial growth; but not for Dilution (*p* = 0.157), or AgNPs@Cs: dilution (p = 0.115). This suggested that there was a significant difference in mean CFU counts among different antimicrobial agents for MRSA. But, for *Pseudomonas aeruginosa*, the p-value was significant for AgNPs@Cs (*p* = 0.000192), and AgNPs@Cs: dilution (*p* = 0.0585), but not for dilution alone (*p* = 0.365). All the previous results suggested that there was a significant difference in mean CFU counts among the different antimicrobial agents, and their interaction with dilution for *Pseudomonas aeruginosa*.

The AgNPs@Cs were used with a starting concentration of 39 µg/ml, which was then serially diluted to concentrations 1.225 µg/ml. All the concentrations; except 1.225 µg/ml; were effective in killing the tested bacterial species, so, we started to test the effect of the photothermal therapy on the least ineffective concentrations; 1.225 µg/ml for AgNPs@Cs, and 1.5 µg/ml for AgNPs. The used antimicrobial agents and the time-point also had a significant interaction effect (*p*-value = 0.0013561), suggesting that the effect of the antimicrobial agent varied with the exposure time to the photo-thermal therapy. The interaction between bacterial species, the antimicrobial agent, and the time point wasn’t statistically significant (*p*-value = 0.0856), indicating that the combined effect of all these three factors wasn’t significant at the conventional significance level of 0.05. Further comparative studies between various Gram-negative and Gram-positive bacteria are required to confirm this hypothesis.

## Conclusion

The small extremely stable AgNPs could be prepared via the two methods chemically, and eco-friendly methods by chitosan as reducing and capping agent which show high stability with zeta potential − 28 mV and 47 mV, respectively. The present study is the first to prepare silver nanoparticles by using chitosan as a reducing and a capping agent in combination with low power laser on bacteria. AgNPs exhibited a photothermal therapeutic effect on the Gram positive (MRSA), along with Gram negative bacteria (*Klebsiella pneumoniae*, and *Pseudomonas aeruginosa*) by combining AgNPs with low power diod laser light (405 nm). That might be suitable to treat the fast-growing drug-resistance bacteria with the slow progress of innovative antimicrobial agents. Furthermore, the assessment of laser low-power radiation coupled with a low dose of AgNPs has a promising effect on the tested bacterial species. The obtained data revealed that the dominant effect was coming from AgNPs by chitosan which were effective and stable in combination with laser, against all the tested microbes. Future work in progress to use the prepared nanoparticles for invivo study and as antibiotic drug carriers against more bacterial species.

## Data Availability

The datasets used and/or analyzed during the present study were obtainable from the corresponding author on reasonable request.
